# Antioxidant system response, mineral element uptake and safe utilization of *Polygonatum sibiricum* in cadmium-contaminated soil

**DOI:** 10.1038/s41598-021-97998-7

**Published:** 2021-09-21

**Authors:** Yuchen Kang, Li Yang, Haibo Dai, Mengdi Xie, Yuhao Wang, Jie Peng, Hui Sun, Tianqi Ao, Wenqing Chen

**Affiliations:** 1 College of Architecture and Environment, Sichuan University, No. 24, South Section 1, 1st Ring Rd., Chengdu, 610065 Sichuan Province China; 2 State Key Laboratory of Hydraulics and Mountain River Engineering, Sichuan University, Chengdu, China; 3 College of Water Resource and Hydropower, Sichuan University, Chengdu, China

**Keywords:** Plant stress responses, Plant physiology, Environmental chemistry

## Abstract

Chinese herbal medicine is widely cultivated in Southwest China, where the soil cadmium (Cd) contamination of farmland is more serious than that in China as a whole. In this study, *Polygonatum sibiricum* was exposed to Cd at concentrations of e^−1^, e^0^, e^2^, and e^4^ mg/kg for 30, 60, and 90 days, and the physiological stress responses, Cd and mineral element uptake, antioxidant enzyme activities, and content changes of pharmaceutical ingredients (polysaccharides) were analyzed to decipher the feasibility of safe utilization in Cd-contaminated soil. The results show that the activity of antioxidant enzymes (SOD and CAT) in the aboveground part was always higher than that in the underground part. The underground part of *Polygonatum sibiricum* mobilizes nonenzymatic systems to facilitate the synthesis of polysaccharides (PCP1, PCP2) with antioxidant properties to cope with Cd stress. Mineral elements (P, K, Ca, Mg, Fe, Cu, and Zn) significantly (*p* < 0.05) changed after 90 d of cultivation. In particular, the changes in the iron and zinc content were significantly correlated (*p* < 0.05) with the activities of SOD and POD. Soil Cd at e^0^ mg/kg can guarantee the safe production and utilization of *Polygonatum sibiricum*, and the stimulation of Cd promotes polysaccharide synthesis and biomass growth.

## Introduction

Southwest China is one of the main production areas of Chinese herbal medicine^1^, but the region has a long history of discharge of Cd-containing wastewater from zinc smelting and waste dumping, and the application of phosphate fertilizers with high Cd content, resulting in compound heavy metal soil pollution that is particularly prominent^2^. Among the various methods to remediate contaminated soil, phytoremediation uses hyperaccumulation to extract heavy metals from the soil. Although this method to eliminate heavy metals or other hazardous chemicals is more eco-friendly than other physical and chemical soil remediation techniques and does not cause secondary soil contamination^3^, it is difficult to promote phytoremediation techniques due to the long restoration cycle and the fact that the economic benefits of cultivated land cannot be guaranteed during the restoration cycle^4^. The purpose of agricultural land soil remediation is to ensure the safety of production and utilization of agricultural products^5^. The cultivation of plants with high economic value and low heavy metal accumulation could solve the current dilemmas of plant remediation and has great application potential.

*Polygonatum sibiricum* is a traditional Chinese herbal medicine of the family *Liliaceae*^6^. This plant is widely distributed in Asia and grows on hillsides or under shaded forests at an altitude of 800–2800 m. The rhizome of *Polygonatum sibiricum* is the main medicinal part, which is a thick block with long sections and a yellowish-brown surface. Polysaccharides in the rhizome of *Polygonatum sibiricum* are the main medicinal component and have been shown to have multiple biological activities including anti-diabetes, anti-inflammation, antioxidant, immune-modulating, and anticancer activities^7,8^. In preliminary studies^9^, we found that the Chinese herbal medicine *Polygonatum sibiricum* has the characteristic of low accumulation of Cd, and because it is rich in polysaccharides and other medicinal active components, it has a high added value and is advantageous for safe cultivation of Cd-contaminated soil. For "low accumulation" cash crops, the antioxidant system is the main mechanism of resistance to heavy metal stress^10^. Heavy metals induce oxidative stress by generating free radicals and reactive oxygen species (ROS), which can interact with lipids, proteins, pigments, and nucleic acids, leading to lipid peroxidation and damage to cell membranes, impairing cellular physiology and the ability to adapt to the environment^11^. The harmful effects of the oxidative state of cells can be mitigated by enzymatic and nonenzymatic antioxidant effects in plants^12^. Superoxide dismutase (SOD), catalase (CAT), and peroxidase (POD) are representative antioxidant enzymes that can scavenge excess ROS produced in plants^13^. SOD dismutates superoxide anions into H_2_O_2_ and O_2_. H_2_O_2_ is destructive to many enzymes and can be degraded to H_2_O and O_2_ by CAT and POD^14^. Nonenzymatic antioxidant systems include β-carotene, α-tocopherol, ascorbic acid, glutathione, and flavonoids, which have certain antioxidant value^15^. Numerous studies have shown that polysaccharides, especially heteropolysaccharides containing proteins and phenols, also have antioxidant properties^16–18^. Therefore, this research investigated polysaccharides, the main material to evaluate the economic value of Chinese herbal medicine^19^, in the nonenzymatic antioxidant system.

Several studies have found that Cd has the potential to induce the synthesis of plant metabolites^20^, including certain pharmacologically active substances with antioxidant properties. *Phyllanthus amarus* under moderate chromium (Cr) stimulation produced more of the therapeutically active secondary metabolites phyllanthin and hypophyllanthin^21^. A similar phenomenon occurs with another medicinal plant, *Vaccinium corymbosum,* in which its antioxidant response is activated, leading to an increase in phenolic compounds under Cd stress^22^. However, this “incentive effect” is not costless. Medicinal plants may lose the ability to synthesize active ingredients at high concentrations of heavy metals; seedlings of St. John's wort completely lose the ability to synthesize or accumulate hyperforin, and the concentrations of pseudohypericin and hypericin demonstrate a 15- to 20-fold decrease^23^.

The mechanism of Cd tolerance in plants with low accumulation is rarely studied, and the responses of their antioxidant active components to Cd are unclear, which calls into question how to ensure the safe use of plants with low accumulation in Cd-contaminated soils. Plant mineral element uptake and tolerance for Cd vary between species, plant growth phases, and soil Cd concentrations; hence, it is necessary to systematically study the behavior of low-accumulation plants under Cd stress. This study was designed to (1) determine the physiological stress responses and interactions between enzymatic and nonenzymatic antioxidant systems of *Polygonatum sibiricum* under Cd stress; (2) analyze Cd and mineral element uptake and polysaccharide content changes in *Polygonatum sibiricum* under Cd stress; and (3) explore the potential of safe utilization of *Polygonatum sibiricum* grown in Cd-contaminated soil.

## Materials and methods

### Plant material and growth conditions

The experiments were conducted with soil medium, and the *Polygonatum sibiricum* was grown in 35 cm × 35 cm × 20 cm wooden pots, each with a soil mass of 15 kg. The experimental soil for potted plants was collected from the Soil Contamination Remediation Project site in Mianzhu, Sichuan Province, with 20% (volumetric ratio) humus and 5% (mass ratio) sulfuric acid-type NPK fertilizer added (total Cd 0.12 mg/kg; available Cd 0.029 mg/kg; pH 7.4). Cd in the experimental soil was added in the form of CdCl_2_-2.5H_2_O, and the designed soil Cd concentration gradients were CK: 0 mg/kg, e^−1^: 0.37 mg/kg, e^0^: 1 mg/kg, e^2^: 7.39 mg/kg, and e^4^: 54.60 mg/kg. Two-year-old seedlings of *Polygonatum sibiricum* were harvested from a traditional Chinese medicine cultivation base in Neijiang City, Sichuan Province. Transplanting is usually carried out after the end of the reverse seedling stage. Therefore, the collected 2-year-old seedlings of Polygonatum sibiricum had no aboveground parts, only underground rhizomes and roots. The rhizome of biennial Polygonatum sibiricum is cylindrical, with enlarged nodules approximately 6–8 cm long, 1–2 cm wide and 2–3 cm thick and average weights of 30–50 g, and the epidermis is yellowish brown. A few roots are distributed around the rhizome. The collection of *Polygonatum sibiricum* complied with guidelines in Sichuan Province and regulations in China. The plants were transplanted to pots after two weeks of soil equilibration and then exposed to Cd stress. From each pot, three plants were randomly collected after 30, 60, and 90 d of cultivation to measure the plant biomass (dry weight), Cd content and mineral element uptake, and polysaccharide content, and the other three plants were collected for the measurement of the antioxidant system parameters. The roots of all plants were soaked in 0.01 mol/L EDTA-2Na solution for 10 min to remove heavy metal ions and precipitates adsorbed on the surface.

### Measurement of the Cd and mineral element content

The dried root, rhizome, stem, and leaf samples were weighed to 0.1 g using an analytical balance. Samples were placed in a crucible with 10 ml of HNO_3_ and 2 ml of HClO_4_ overnight, digested on an electric plate until nearly dry and transferred to a 15 ml centrifuge tube, which was fixed with 1% HNO_3_ to 15 ml. Samples were analyzed by inductively coupled plasma mass spectrometry (SHIMADZU ICPE-9000, JPN).

### Enzyme and polysaccharide antioxidant activity analysis

The activity of SOD was determined according to the method of Jia et al.^24^. The activities of CAT and POD were evaluated using the improved methods by Azevedo et al.^25^. The pyrogallol autoxidation method was used according to Zhang et al.^26^ to determine the antioxidant activity of polysaccharides.

### Extraction of polysaccharides

Using the graded extraction method, 0.1 g of dried flavin was taken, and the residue was degreased by refluxing at 80 °C for 24 h. The residue was dried to obtain the defatted flavin sample. The sample was decocted in 10 mL of distilled water for 2 h each time and sonicated for 1 h. The filtrate was filtered, combined, and transferred to a 50 ml flask, where the liquid polysaccharide sample was PCP1. Taking the first stage of filtration and adding 0.1% NaOH solution to extract, the same steps as above were repeated to acquire the polysaccharide sample called PCP2. The second filtrate was extracted by adding 0.5% NaOH solution, repeating the same steps as above to obtain the polysaccharide sample named PCP3.

### Determination of polysaccharide content and molecular weight

The glucose solution was dried to a constant weight (105 °C), then 33 mg was taken and transferred to a 100 ml flask. In an ice water bath, 0.2% anthrone—sulfuric acid solution was slowly added to the scale, mixed well and cooled for 10 min in a 100℃-water bath, then immediately put it in an ice-water bath. The absorbance at 582 nm was measured by a UV–Vis spectrophotometer (MAPADA UV-6100S, CHN) for 10 min. The polysaccharide liquid sample was treated as described above, the absorbance was measured, and the polysaccharide content was calculated against the standard curve. The polysaccharide molecular weight was determined by high-performance gel permeation chromatography according to Peng et al.^27^.

### Statistical analysis

Collection and aggregation of raw data were performed using Excel, and mapping was conducted using Origin 9.0 software. Data for the biomass, Cd and mineral element content, enzyme activities, and content of polysaccharide were subjected to correlation analysis, one-factor ANOVA, and Duncan’s multiple test using GraphPad Prism 8.0 software.

### Ethics approval and consent to participate

Not applicable.

### Consent for publication

Not applicable.

### Statement in the collection of plant material

The collection of *Polygonatum sibiricum* is in compliance with guidelines in Sichuan province and regulations in China. All collection was done with the permission of the relevant regulatory governing bodies and with reference to the relevant legislation.

## Results and discussion

### Biomass and plants height

**Figure 1:**
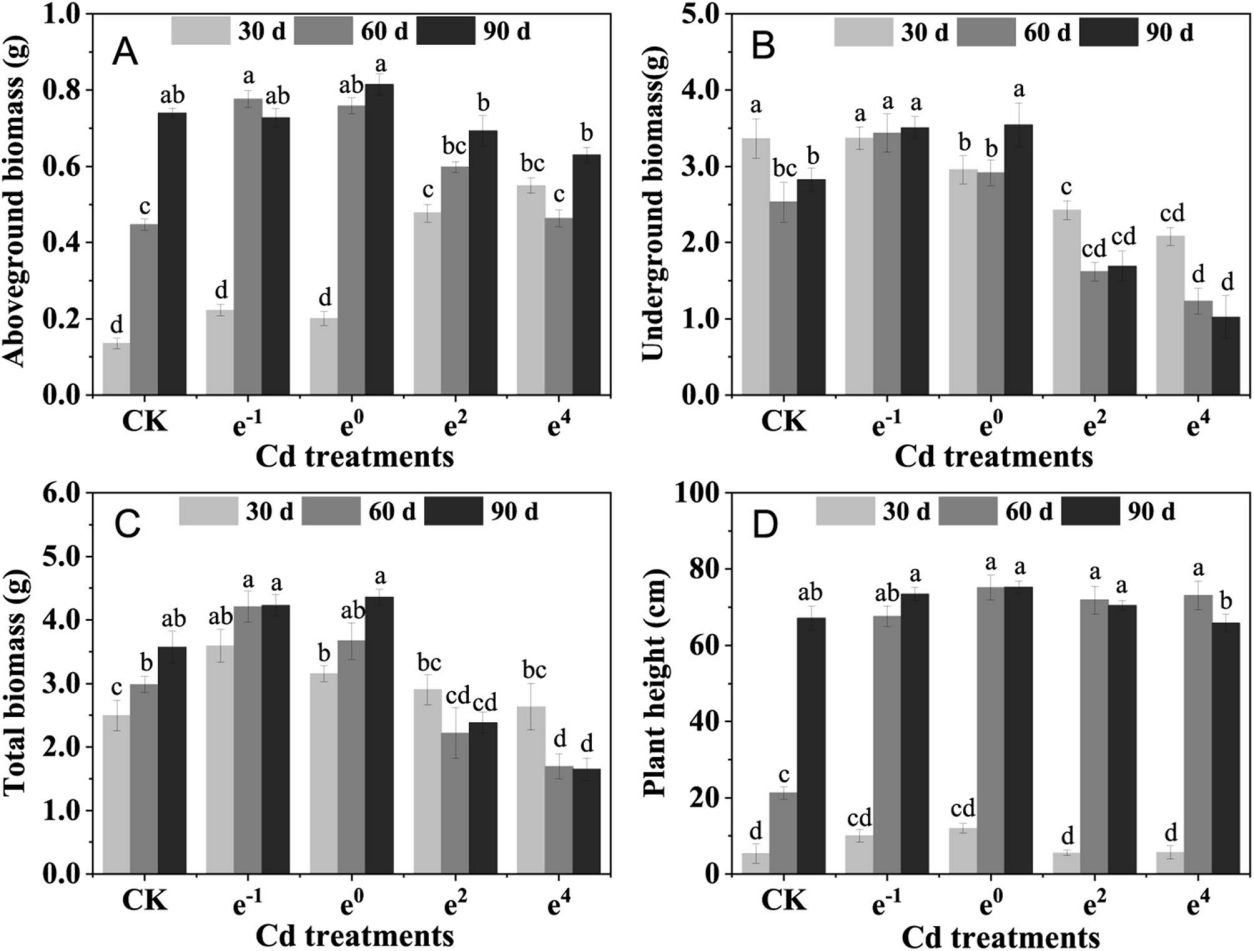
The biomass of *Polygonatum sibiricum* under different Cd stresses. (**A**) Aboveground biomass (including roots and rhizomes); (**B**) Underground biomass (including stems and leaves); (**C**) Total biomass; (**D**) Plant height. Each value represents the mean ± standard deviation of three independent experiments. Different letters above the bars represent significant differences (*p* < 0.05).

The aboveground (stems and leaves) biomass and underground (roots and rhizomes) biomass showed opposite changes after 30 d of cultivation (Fig. 1A,B): the biomass of underground parts was smaller than that of the control group whereas the biomass of aboveground parts showed a growth trend as the Cd concentration increased. However, a significantly (*p* < 0.05) negative influence of biomass was observed in the e^2^ and e^4^ treatments after 90 d of cultivation. In comparison to the CK treatment, the biomass of the e^2^ and e^4^ groups significantly (*p* < 0.05) decreased by 40.22% and 63.90% (underground biomass) and by 33.27% and 53.85% (total biomass), respectively. After 90 days of cultivation, the biomass of the underground part and total plants exposed to the e^−1^ and e^0^ treatments significantly (*p* < 0.05) increased by 24.03% and 25.41% (underground biomass) and by 18.66% and 22.23% (total biomass), respectively, compared with the CK treatment (Fig. 1B,C). The total biomass (Fig. 1C) and plant height (Fig. 1D) showed significantly (*p* < 0.05) stable growth in the e^−1^ and e^0^ treatment treatments after 90 days of cultivation. This phenomenon indicates that soil Cd at concentrations of e^−1^ and e^0^ mg/kg has positive effects on the growth of the plant and that *Polygonatum sibiricum* exhibited good tolerance to Cd during persistent interaction with Cd in the soil.

### Cd content in different parts of plants

**Figure 2:**
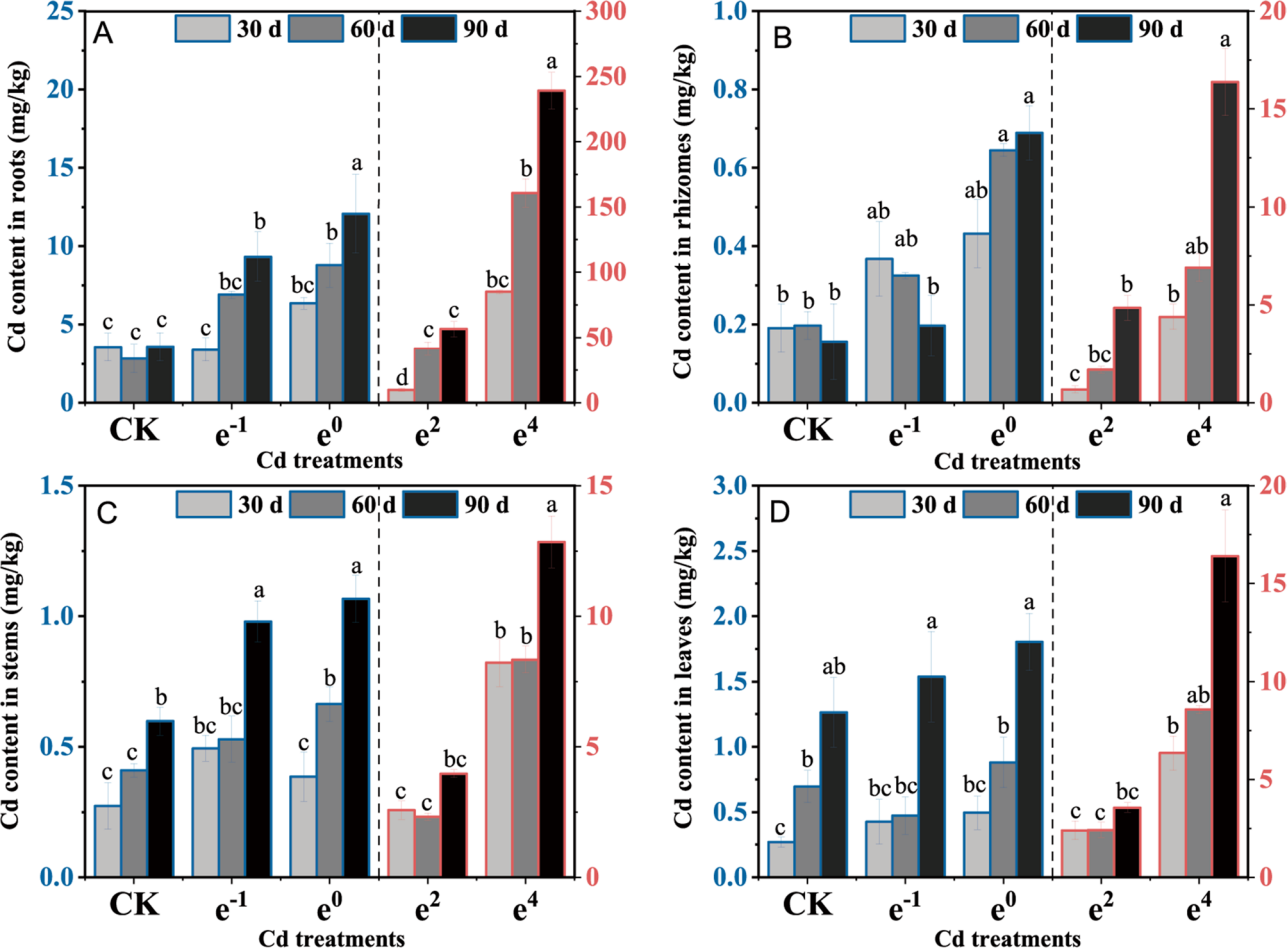
Cd content in the roots, rhizomes, stems, and leaves of *Polygonatum sibiricum*. (**A**) Root Cd content; (**B**) rhizome Cd content; (**C**) stem Cd content; (**D**) leaf Cd content. Each value represents the mean ± standard deviation of three independent experiments. Different letters above the bars represent significant differences (*p* < 0.05).

The Cd levels in plants increased in a dose-dependent manner (Fig. 2). The highest Cd content occurred in the e^4^ treatment after 90 d of cultivation, under which condition the Cd content was 239.04, 16.38, 12.84, and 16.41 mg/kg in the roots, rhizomes, stems, and leaves, respectively. The root Cd content was higher than that in other parts in all treatments and significantly (*p* < 0.05) increased with cultivation time (Fig. 2A). The Cd content in the medicinal site rhizome of 0.36 (30 d, e^−1^), 0.43 (30 d, e^0^), 0.33 (60 d, e^−1^), 0.64 (60 d, e^0^), 0.20 (90 d, e^−1^), 0.69 (90 d, e^0^) mg/kg (Fig. 2B) was lower than the limit for Cd in the Pharmacopoeia of the People's Republic of China, but it failed to meet the requirements in the e^2^ and e^4^ treatments due to an excessive soil Cd concentration. After 90 d of cultivation, the Cd content in stems and leaves was significantly (*p* < 0.05) higher than that at 30 d of cultivation and increased as Cd levels increased (Fig. 2C,D). Previous studies have shown that the roots could have the highest Cd content in plants^28,29^ because roots are the primary organs in the response to Cd stress in soil, and Cd can complex with proteins, cellulose or pectates or insoluble Cd phosphate in the root cell wall^30^. This characteristic of Cd uptake in roots is consistent with the accumulation of heavy metals in root-hoarding plants. Root-hoarding plants store heavy metals mainly in the roots, and only a small amount of heavy metal is transferred to the ground, which reduces damage to the photosynthetic, respiratory, and reproductive systems^31^. This "root-retention" characteristic of *Polygonatum sibiricum* is beneficial to improve survivability in Cd-contaminated soil and ensure the safety of medicinal parts.

### Antioxidant enzyme system

**Figure 3:**
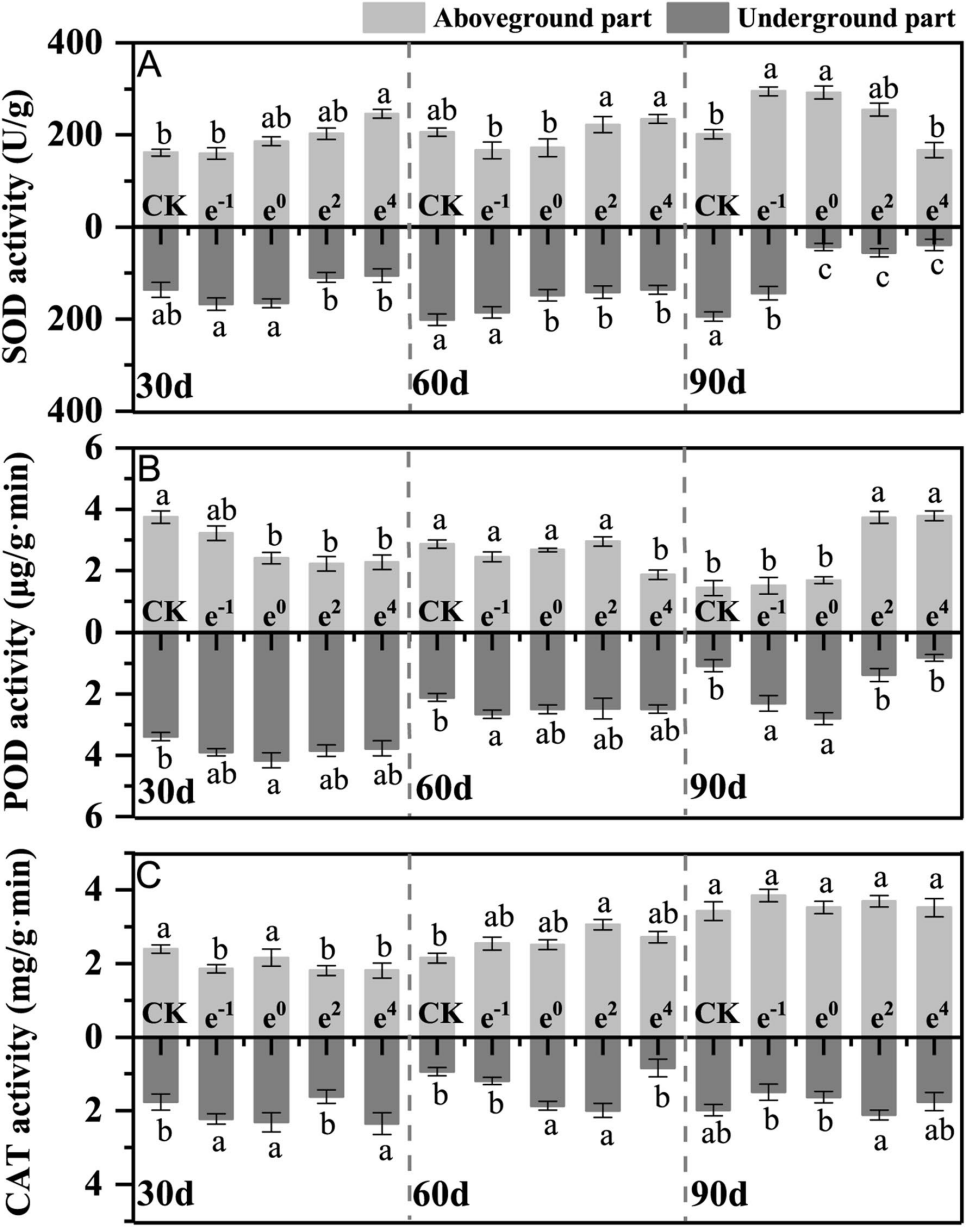
Antioxidant enzyme activity of *Polygonatum sibiricum*. (**A**) Superoxide dismutase (SOD) activity in the aboveground and underground parts; (**B**) Peroxidase (POD) activity in the aboveground and underground parts; (**C**) Catalase (CAT) activity in aboveground and underground parts. Each value represents the mean ± standard deviation of three independent experiments. Different letters above the bars represent significant differences (*p* < 0.05).

The aboveground and underground parts showed different patterns of SOD and POD activity (Fig. 3A–B). In the aboveground part after 30 d of cultivation, the SOD activity significantly (*p* < 0.05) increased with increasing Cd levels, reaching a maximum in the e^4^ treatment that was 52.17% higher than the control group. After 90 d of cultivation, the SOD activity was 1.47, 1.45, and 1.27 times higher than that of CK in the e^−1^, e^0^, and e^2^ treatments, respectively. However, for the underground parts, the higher Cd treatment showed lower SOD activity throughout the full cultivation time. Especially after 90 d of cultivation, the SOD activity of the underground part decreased significantly (*p* < 0.05) by 77.69%, 71.31% and 79.99% in the e^0^, e^2^ and e^4^ treatments, respectively, compared with the CK treatment. Furthermore, significant negative relationships were found between the Cd content and SOD activity in the underground part (r = −0.5538, *p* < 0.05) (Table 1), indicating that the response of SOD to Cd was suppressed slightly. The POD activity of aboveground/underground parts significantly (*p* < 0.05) increased/decreased under Cd treatments compared with the CK treatment after 30 d of cultivation. After 90 d of cultivation, the aboveground POD activity was 6.41 and 6.47 times higher than that of the CK treatment in the e^2^ and e^4^ treatments, respectively. For the underground part, the POD activity was slightly altered in response to Cd stress after 30 and 60 d of cultivation, and increased significantly (*p* < 0.05) by 113.89% and 159.26% in the e^−1^ to e^0^ treatments, respectively, compared with the CK treatment. However, the overall level of POD activity in the underground part decreased with the cultivation time. As shown in Fig. 3C, the CAT activities in the aboveground part increased as the cultivation time increased, and the aboveground enzyme activity was higher than that of the underground part.

**Table 1:** Pearson correlation coefficients of the Cd content, enzyme activity, polysaccharide content, and mineral element content of *Polygonatum sibiricum*.

	Cd	SOD	POD	CAT	PCP1	PCP2	PCP3	TPCP	P	K	Ca	Mg	Fe	Cu	Zn
Cd	1.0000														
SOD	− 0.5538*	1.0000													
POD	− 0.4012	0.0862	1.0000												
CAT	− 0.0138	− 0.0610	0.1784	1.0000											
PCP1	− 0.4740	0.1002	0.8394*	0.2836	1.0000										
PCP2	− 0.0638	0.1568	− 0.2821	0.1527	− 0.3403	1.0000									
PCP3	− 0.2189	0.2496	0.3131	0.3440	0.2144	0.7041*	1.0000								
TPCP	− 0.5100	0.1595	0.8231*	0.3528	0.9595*	− 0.0693	0.4666	1.0000							
P	− 0.1112	0.4149	− 0.3026	0.1343	− 0.1328	0.4595	0.2219	− 0.0214	1.0000						
K	− 0.2410	− 0.1784	− 0.3484	0.2251	− 0.3657	0.3415	0.0653	− 0.2938	− 0.1942	1.0000					
Ca	0.1032	− 0.1035	− 0.6087*	0.0338	− 0.5349*	0.4622	− 0.0922	− 0.4604	0.4973	0.2525	1.0000				
Mg	− 0.0201	0.3260	− 0.5664*	0.0535	− 0.3922	0.2588	− 0.1074	− 0.3569	0.7433*	− 0.0418	0.4646	1.0000			
Fe	0.7613*	− 0.7291*	− 0.6349*	− 0.1977	− 0.6956*	0.0574	− 0.3919	− 0.7306*	− 0.0446	0.1981	0.4085	0.1253	1.0000		
Cu	0.6337*	− 0.4306	− 0.4328	0.2421	− 0.4165	0.3456	0.1435	− 0.3335	0.4643	0.0314	0.3524	0.3148	0.6654*	1.0000	
Zn	0.6320*	− 0.5768*	− 0.7501*	0.1682	− 0.6445*	0.1792	− 0.2666	− 0.6420*	0.0447	0.3662	0.5506*	0.3056	0.8199*	0.5671*	1.0000

*p < 0.05.

Typically, studies of plant antioxidant enzyme activity have focused on the aboveground part, with few experiments considering the differences between aboveground and underground antioxidant enzymes. The aboveground SOD activity of *Polygonatum sibiricum* was similar to that of most plants, but the SOD activity of the underground parts was lower than that of the control group under a higher Cd level (e^0^, e^2^, and e^4^ treatment). The results show that the response thresholds of SOD, POD, and CAT to Cd stimulation were different, and the correlation between the effect of Cd stimulation on the activities of antioxidant enzymes and the concentration of Cd in plants was always variable. Some researchers suggest that Cd inhibits the activity of antioxidant enzymes^32^, and some show that Cd stress could activate antioxidant enzymes^33^. Other studies indicate the aboveground and underground parts of the same plant have different responses to antioxidant enzyme activity^34^. Here, the changes in CAT and POD activity were not uniform, which indicates that antioxidant enzyme activities are related to the plant species, and different tolerance behaviors are exploited behavior to alleviate Cd-induced oxidative stress.

### Mineral element uptake

**Figure 4:**
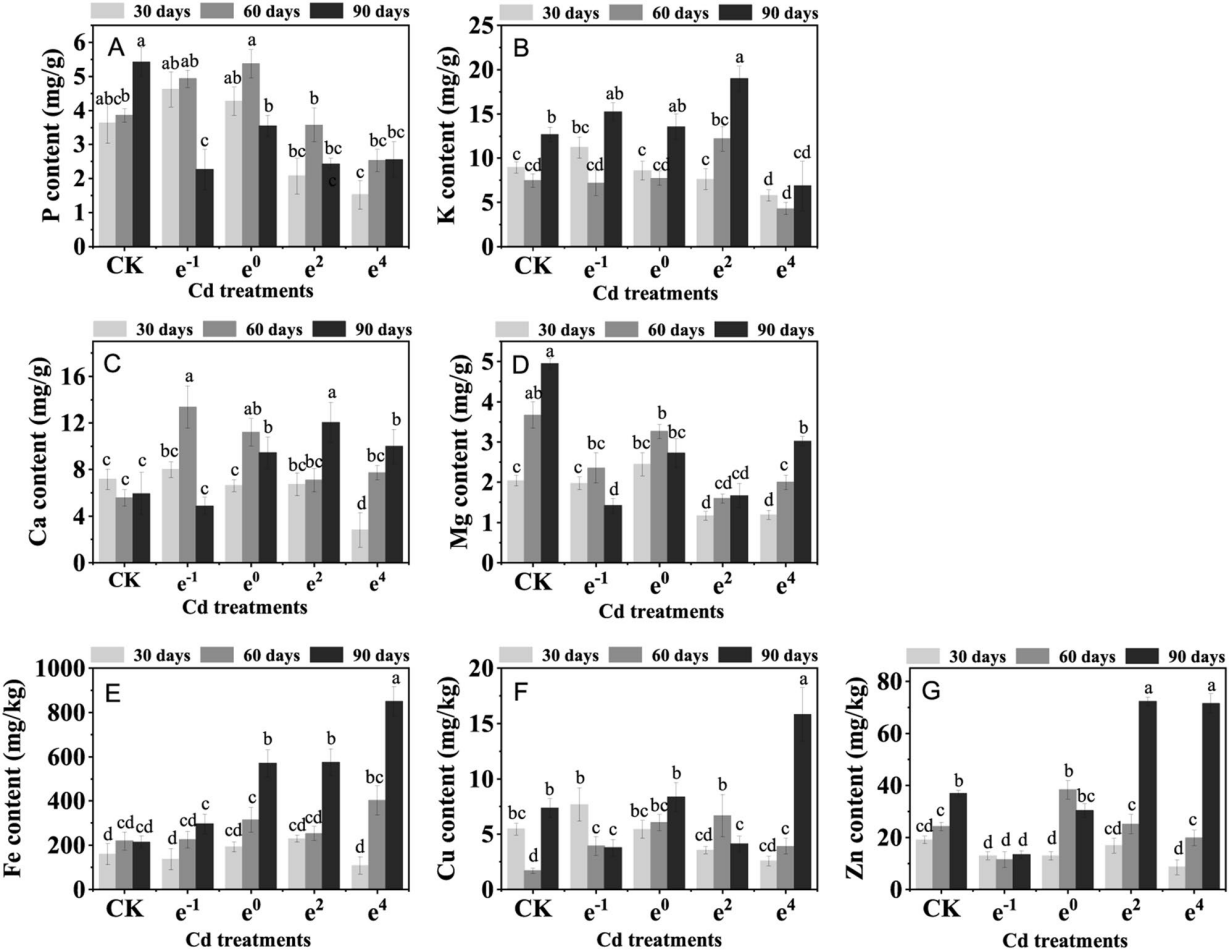
Changes in the P, K, Ca, Mg, Fe, Cu, and Zn content of *Polygonatum sibiricum* under Cd stress. **A, B, C, D, E, F**, and **G** represent P, K, Ca, Mg, Fe, Cu, and Zn, respectively. Each value represents the mean ± standard deviation of three independent experiments. Different letters above the bars represent significant differences (*p* < 0.05).

The changes in macronutrient levels (P, K, Ca, and Mg) in *Polygonatum sibiricum* in response to Cd stress are shown in Fig. 4A–D. Phosphorus (P) is an essential macronutrient that supports plant growth and reduces the toxicity of cadmium by chelating or forming complexes with cadmium in plants, thereby reducing the damage to cell function caused by Cd^35^. In the e^−1^ and e^0^ treatments, the P content was significantly (*p* < 0.05) increased by 27.61% and 17.72%, respectively, after 30 days and 27.93% and 39.32%, respectively, after 60 d of cultivation compared to the CK treatment. However, under higher Cd stress (e^4^) and long-term Cd stress for 90 d, the P content significantly (*p* < 0.05) increased. This indicates that Cd can affect the uptake and accumulation of elemental P in *Polygonatum sibiricum,* while P was described as having no effect on Cd uptake^36^.

Potassium (K) is the most abundant inorganic cation in plant cells^37^. The K content in all treatments reached a maximum value after 90 d of cultivation and, to varying degrees, showed a facilitative effect of Cd on K uptake except for in the e^4^ treatment. This phenomenon might be related to the ability of Cd to increase the influx of K^+^ ions by binding to K channels and opening them permanently^38,39^; the complexation of ATP with Cd proved that the absorption of K decreased and the available energy of the membrane transport system decreased, leading to disruption in the plasma membrane and causing the decline of K under Cd concentrations as a result of K leakage^40^.

The calcium (Ca) content was significantly (*p* < 0.05) promoted by Cd stress and increased by 140.03%, 101.25%, 27.11%, and 38.35% in the e^−1^, e^0^, e^2^, and e^4^ treatments, respectively, after 60 d of cultivation. However, after 90 d of cultivation the Ca uptake was inhibited except in the e^2^ treatment. It has been reported that the Ca content in plants growing in Cd-contaminated solutions is reduced in different species, possibly due to competition between Cd^2+^ and divalent cations during the absorption process^41,42^. However, studies have also shown that the action of Cd on Ca channels and transporter proteins leads to an increase in their transcription and translation, thus allowing for greater Ca uptake and compensating for the blocking effect of Ca channels^38^. Thus, the interactions between Ca and Cd are adjusted according to the concentration of Cd and the duration of stress.

The magnesium (Mg) content decreased progressively with increasing plant cultivation time. Compared with the CK treatment after 90 d of cultivation, the Mg content significantly (*p* < 0.05) declined by 71.46%, 45.05%, 66.26%, and 38.99% in the e^−1^, e^0^, e^2^, and e^4^ treatments, respectively. The Pearson correlation coefficients between the Mg content and POD activity (r =  − 0.5664, *p* < 0.05) (Table 1) indicate that the toxicity of Cd can promote the reduction of Mg, which affects the enzyme activity because Mg is a master activator of more than 300 enzymes^43^.

In this study, significant positive relationships were found between the Cd content and iron (Fe), copper (Cu) and zinc (Zn) content (r = 0.7613, 0.6337 and 0.6320, *p* < 0.05). Moreover, the content of Fe, Cu and Zn are strongly correlated (r = 0.6654, 0.8199 and 0.5671, *p* < 0.05). After 90 d of cultivation, the Fe, Cu, and Zn content increased under the high-Cd treatment compared with the CK treatment (Fig. 4E–G). The Fe and Zn content were strongly negatively correlated with the SOD and POD activity (r = −0.7291 and −0.5768, −0.6349 and −0.7501, *p* < 0.05, respectively) and PCP1 and TPCP content (r = −0.6956 and −0.6445, −0.7306 and −0.6420, *p* < 0.05, respectively). Fe, Cu, and Zn form enzymes that are crucial in plant antioxidative mechanisms, and Cd replaces/displaces Fe, Cu, and Zn in enzymes or other molecules with different macromolecules. Thus, this effect may plunge regulatory mechanisms into a state of Fe/Cu/Zn deficiency, leading to an increase in their uptake as an overcompensatory mechanism^44^. The toxicity of Cd to plants disrupts the uptake and distribution of mineral elements in tissues, leading to mineral deficiencies, overcompensation, or imbalance, which affect the activity of related enzymes and cause damage to the plant's antioxidant system.

### Polysaccharide content and its antioxidant properties

**Figure 5:**
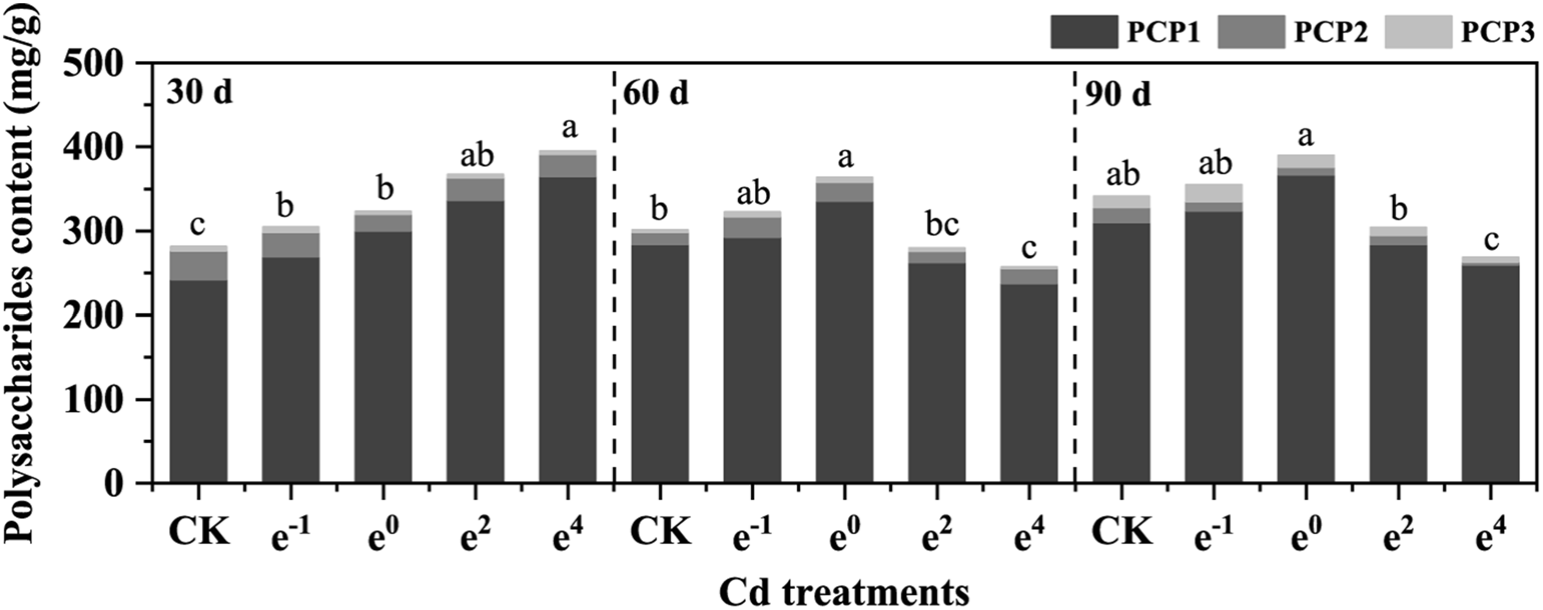
Polysaccharide content of *Polygonatum sibiricum.* PCP1: First step polysaccharides in fractionated extraction. PCP2: Second step polysaccharides in fractionated extraction. PCP3: Third step polysaccharides in fractionated extraction.

Compared with the control group, *Polygonatum sibiricum* was appropriately stimulated to increase the polysaccharide content in all treatments through 30 d of cultivation. At a higher Cd level, this stimulatory effect was reduced, as evidenced by the inhibition of polysaccharide synthesis in the e^4^ treatment similar to after 60 and 90 d of cultivation (Fig. 5). However, the total polysaccharides after 90 d of cultivation decreased by 8.45%, 20.25%, 46.12%, and 50.77% in the four treatments compared with that at 30 d of cultivation. However, it is worth noting that the control group decreased by 16.31%. The depletion of polysaccharides in rhizomes is presumed to be due to the growing period as well as to excessive Cd stress. Among them, Cd stress showed the best promotion effect on polysaccharide synthesis in the e^0^ treatment.

The antioxidant activity of the three polysaccharides in the rhizome of *Polygonatum sibiricum* was in the order of PCP1 > PCP2 > PCP3 (Table 2). The polysaccharides from the first step and second step showed superoxide anion scavenging rates of 5.61% and 3.06%, respectively. The polysaccharides from the last step did not show antioxidant activity. Evidence has proven that the molecular weight distributions of polysaccharides greatly influence their biological activities^45^. PCP1 has the lowest molecular weight and the best performance in scavenging superoxide radicals, which could indicate a similar result: high-molecular-weight polysaccharides are less active than low-molecular-weight polysaccharides^46^.

**Table 2:** The clearance rate of ·O_2_^-^ and molecular weight of polysaccharides in *Polygonatum sibiricum.*

Polysaccharides	Scavenging rate (%)	Molecular weight (Mw/Da)	Molecular weight (Mn/Da)
PCP1	5.61 ± 0.98a	26,345	931
PCP2	3.06 ± 0.57b	51,557	557
PCP3	0.00 ± 3.39c	177,316	755

Saccharides are a source of nutrients and a component of the structural parts of plants, but an increasing number of studies show that sugars play an important role in plant stress tolerance^47,48^. Most of the polysaccharides in plants are heteropolysaccharides, which consist of various kinds of monosaccharides as well as proteins and phenols. The antioxidant functional groups of these substances can significantly enhance the antioxidant properties of plant polysaccharides. Therefore, the role of polysaccharides as nonenzymatic antioxidants in plant stress tolerance cannot be ignored. In the correlation analysis (Table 1), there was a significant positive correlation (r = 0.8394, *p* < 0.01) between the polysaccharides and POD activity. Few studies have investigated the role of polysaccharides as part of a nonenzymatic antioxidant system in plant resilience, but several studies have shown that plant polysaccharides have antioxidant effects and mitigate heavy metal toxicity^49–51^.

## Conclusion

In the e^−1^ and e^0^ treatments, the Cd content of the *Polygonatum sibiricum* rhizome met the consumption standard for heavy metals in Chinese herbal medicine stipulated in the Pharmacopoeia of the People's Republic of China, and the biomass of *Polygonatum sibiricum* increased, showing Cd tolerance and utilization safety. For the aboveground part of *Polygonatum sibiricum*, the SOD and CAT activities increased to cope with the ROS generated by oxidative stress at higher Cd concentrations. For the underground part, enzymatic and nonenzymatic systems act synergistically, resulting in an enhancement in antioxidant enzyme activity and an increase in polysaccharide synthesis at lower Cd treatment. Both enzymatic and nonenzymatic systems were partially inhibited at higher Cd treatments. The stimulatory effect of Cd changes the mineral element uptake of *Polygonatum sibiricum,* especially in the high-Cd treatment, and influences the enzyme system of plants. In conclusion, the safe utilization of *Polygonatum sibiricum* can be guaranteed when the soil Cd concentration is under 1 mg/kg, and it has high application potential in soil remediation areas with lower Cd contamination.

## Data Availability

The datasets used and/or analyzed during the current study are available from the corresponding author on reasonable request.

## References

[1] Kang C-Z (2020). Pattern of ecological planting for Chinese materia medica based on regional distribution. China J. Chin. Mater. Med..

[2] Luo YM, Teng Y (2018). Regional difference in soil pollution and strategy of soil zonal governance and remediation in China. Bull. Chin. Acad. Sci..

[3] Kanwar VS, Sharma A, Srivastav AL, Rani L (2020). Phytoremediation of toxic metals present in soil and water environment: A critical review. Environ. Sci. Pollut. Res..

[4] Suresh B, Ravishankar GA (2004). Phytoremediation—A novel and promising approach for environmental clean-up. Crit. Rev. Biotechnol..

[5] Zhang TL, Wang XX (2019). Prevention and remediation of soil contamination to strengthen the foundation for green and high-quality agricultural development in China. Acta Pedol. Sin..

[6] Chen J (2021). Botrytis cinerea causing gray mold of *Polygonatum sibiricum* (Huang Jing) in China. Crop Prot..

[7] Xie Y (2021). Polysaccharide-rich extract from *Polygonatum sibiricum* protects hematopoiesis in bone marrow suppressed by triple negative breast cancer. Biomed. Pharmacother..

[8] Han C (2020). Protective effect of *Polygonatum sibiricum* against cadmium-induced testicular injury in mice through inhibiting oxidative stress and mitochondria-mediated apoptosis. J. Ethnopharmacol..

[9] Teng, S.-R. *Effects of Exogenous Se on Growth and Internal Quality of Polygonatum sibiricum*, Hubei Minzu University (2018).

[10] Maleki M, Ghorbanpour M, Kariman K (2017). Physiological and antioxidative responses of medicinal plants exposed to heavy metals stress. Plant Gene.

[11] Dissanayake NM, Current KM, Obare SO (2015). Mutagenic effects of iron oxide nanoparticles on biological cells. Int. J. Mol. Sci..

[12] Arif N (2016). Influence of high and low levels of plant-beneficial heavy metal ions on plant growth and development. Front. Environ. Sci..

[13] Mittler R (2002). Oxidative stress, antioxidants and stress tolerance. TRENDS Plant Sci..

[14] Tewari RK, Kumar P, Sharma PN (2006). Antioxidant responses to enhanced generation of superoxide anion radical and hydrogen peroxide in the copper-stressed mulberry plants. Planta.

[15] Wu F, Zhang G, Dominy P, Wu H, Bachir DML (2007). Differences in yield components and kernel Cd accumulation in response to Cd toxicity in four barley genotypes. Chemosphere.

[16] Ross KA, Godfrey D, Fukumoto L (2015). The chemical composition, antioxidant activity and α-glucosidase inhibitory activity of water-extractable polysaccharide conjugates from northern Manitoba lingonberry. Cogent Food Agric..

[17] Liu J (2018). Isolation, structural characterization and bioactivities of naturally occurring polysaccharide-polyphenolic conjugates from medicinal plants—A reivew. Int. J. Biol. Macromol..

[18] Fu JL (2018). A mechanism by which Astragalus polysaccharide protects against ROS toxicity through inhibiting the protein dephosphorylation of boar sperm preserved at 4°C. J. Cell. Physiol..

[19] Kang C-Z, Zhou T, Jiang W-K, Huang L-Q, Guo L-P (2016). Research model on commodity specification standard of radix Chinese materia medica. China J. Chin. Mater. Med..

[20] Xie MD (2020). Metabolomics reveals the "Invisible" detoxification mechanisms of *Amaranthus hypochondriacus* at three ages upon exposure to different levels of cadmium. Ecotox. Environ. Safe..

[21] Rai V, Mehrotra S (2008). Chromium-induced changes in ultramorphology and secondary metabolites of *Phyllanthus amarus* Schum & Thonn.—An hepatoprotective plant. Environ. Monit. Assess..

[22] Manquian-Cerda K (2016). Effect of cadmium on phenolic compounds, antioxidant enzyme activity and oxidative stress in blueberry (*Vaccinium corymbosum* L.) plantlets grown in vitro. Ecotox. Environ. Safe..

[23] Murch SJ, Haq K, Rupasinghe HPV, Saxena PK (2003). Nickel contamination affects growth and secondary metabolite composition of St. John's wort (*Hypericum perforatum* L.). Environ. Exp. Bot..

[24] Jia X, Zhao YH, Liu T, He YH (2017). Leaf defense system of *Robinia pseudoacacia* L. seedlings exposed to 3 years of elevated atmospheric CO2 and Cd-contaminated soils. Sci. Total Environ..

[25] Azevedo RA, Alas RM, Smith RJ, Lea PJ (1998). Response of antioxidant enzymes to transfer from elevated carbon dioxide to air and ozone fumigation, in the leaves and roots of wild-type and a catalase-deficient mutant of barley. Physiol. Plant..

[26] Zhang QA, Wang X, Song Y, Fan XH, Martin JFG (2016). Optimization of pyrogallol autoxidation conditions and its application in evaluation of superoxide anion radical scavenging capacity for four antioxidants. J. AOAC Int..

[27] Peng Q, Li M, Xue F, Liu HJ (2014). Structure and immunobiological activity of a new polysaccharide from *Bletilla striata*. Carbohydr. Polym..

[28] Jiang Y (2021). Overexpression of SmZIP plays important roles in Cd accumulation and translocation, subcellular distribution, and chemical forms in transgenic tobacco under Cd stress. Ecotox. Environ. Safe..

[29] Rui H, Chen C, Zhang X, Shen Z, Zhang F (2016). Cd-induced oxidative stress and lignification in the roots of two *Vicia sativa* L. varieties with different Cd tolerances. J. Hazard. Mater..

[30] Huang RZ, Jiang YB, Jia CH, Jiang SM, Yan XP (2018). Subcellular distribution and chemical forms of cadmium in *Morus alba* L. Int. J. Phytoremediat..

[31] Lei M, Yuo QL, Chen TB, Huang ZC, Liao XY (2005). Heavy metal concentrations in soils and plants around Shizuyuan mining area of Hunan Province. Acta Ecol. Sin..

[32] Nahakpam S, Shah K (2011). Expression of key antioxidant enzymes under combined effect of heat and cadmium toxicity in growing rice seedlings. Plant Growth Regul..

[33] Dinakar N, Nagajyothi PC, Suresh S, Udaykiran Y, Damodharam T (2008). Phytotoxicity of cadmium on protein, proline and antioxidant enzyme activities in growing *Arachis hypogaea* L. seedlings. J. Environ. Sci..

[34] Zoufan P, Jalali R, Hassibi P, Neisi E, Rastegarzadeh S (2018). Evaluation of antioxidant bioindicators and growth responses in *Malva parviflora* L. exposed to cadmium. Physiol. Mol. Biol. Plants.

[35] Gomes MP, Soares AM, Garcia QS (2014). Phosphorous and sulfur nutrition modulate antioxidant defenses in *Myracrodruom urundeuva* plants exposed to arsenic. J. Hazard. Materi..

[36] Zhong WL, Li JT, Chen YT, Shu WS, Liao B (2012). A study on the effects of lead, cadmium and phosphorus on the lead and cadmium uptake efficacy of Viola baoshanensis inoculated with arbuscular mycorrhizal fungi. J. Environ. Monit..

[37] Benito B, Haro R, Amtmann A, Cuin TA, Dreyer I (2014). The twins K+ and Na+ in plants. J. Plant Physiol..

[38] De la Rosa G (2005). Production of low-molecular weight thiols as a response to cadmium uptake by tumbleweed (*Salsola kah*). Plant Physiol. Biochem..

[39] Mourato, M. *et al.* in *Cadmium Toxicity and Tolerance in Plants* (eds Mirza Hasanuzzaman, Majeti Narasimha Vara Prasad, & Masayuki Fujita) 327–348 (Academic Press, 2019).

[40] Ghnaya T (2005). Cadmium effects on growth and mineral nutrition of two halophytes: *Sesuvium portulacastrum* and *Mesembryanthemum crystallinum*. J. Plant Physiol..

[41] Walker WM, Miller JE, Hassett JJ (1977). Effect of lead and cadmium upon calcium, magnesium, potassium, and phosphorus concentration in young corn plantS. Soil Sci..

[42] Paoli L, Vannini A, Monaci F, Loppi S (2018). Competition between heavy metal ions for binding sites in lichens: Implications for biomonitoring studies. Chemosphere.

[43] Bose J, Babourina O, Rengel Z (2011). Role of magnesium in alleviation of aluminium toxicity in plants. J. Exp. Bot..

[44] Assuncao AGL, Bleeker P, ten Bookum WM, Vooijs R, Schat H (2008). Intraspecific variation of metal preference patterns for hyperaccumulation in *Thlaspi caerulescens*: Evidence from binary metal exposures. Plant Soil.

[45] Sheng JW, Sun YL (2014). Antioxidant properties of different molecular weight polysaccharides from *Athyrium multidentatum* (Doll.) Ching. Carbohydr. Polym..

[46] Wu Y-T (2020). Purification, characterization and antioxidant activity of polysaccharides from *Porphyra haitanensis*. Int. J. Biol. Macromol..

[47] Pirselova B, Matusikova I (2013). Callose: The plant cell wall polysaccharide with multiple biological functions. Acta Physiol. Plant..

[48] Xu SS, Lin SZ, Lai ZX (2015). Cadmium impairs iron homeostasis in Arabidopsis thaliana by increasing the polysaccharide contents and the iron-binding capacity of root cell walls. Plant Soil.

[49] Sun HQ (2020). Function and mechanism of polysaccharide on enhancing tolerance of Trichoderma asperellum under Pb2+ stress. Int. J. Biol. Macromol..

[50] Li SP (2003). A polysaccharide isolated from *Cordyceps sinensis*, a traditional Chinese medicine, protects PC12 cells against hydrogen peroxide-induced injury. Life Sci..

[51] Li JJ (2019). Biochemical changes of polysaccharides and proteins within EPS under Pb (II) stress in *Rhodotorula mucilaginosa*. Ecotox. Environ. Safe..

